# Enumeration of leukocyte infiltration in solid tumors by confocal laser scanning microscopy

**DOI:** 10.1186/1471-2172-7-16

**Published:** 2006-07-21

**Authors:** J Biggerstaff, B Weidow, A Amirkhosravi, JL Francis

**Affiliations:** 1 Biological Imaging Unit, Center for Biomarker Analysis, 10515 Research Drive, Suite 300, Knoxville, TN 37932, USA; 2 Florida Hospital Institute of Translational Research, 2501 N. Orange Avenue, Suite 786, Orlando, FL 32804, USA

## Abstract

**Background:**

Leukocytes commonly infiltrate solid tumors, and have been implicated in the mechanism of spontaneous regression in some cancers. Conventional techniques for the quantitative estimation of leukocyte infiltrates in tumors rely on light microscopy of immunostained thin tissue sections, in which an arbitrary assessment (based on low, medium or high levels of infiltration) of antigen density is made by the pathologist. These estimates are relatively subjective and often require the opinion of a second pathologist. In addition, since thin tissue sections are cut, no data regarding the three-dimensional distribution of antigen can be obtained.

**Results:**

To overcome these problems, we have designed a method to enumerate leukocyte infiltration into tumors, using confocal laser scanning microscopy of fluorescently immunostained leukocytes in thick tissue sections. Using image analysis software, a threshold was applied to eliminate unstained tissue and residual noise. The total antigen volume in the scanned tissue was calculated and divided by the mean cell volume (calculated by "seeding" ten individual cells) to obtain the cell count. Using this method, we compared the calculated leukocyte counts with those obtained manually by ten laboratory personnel. There was no significant difference (P > 0.05) between the cell counts obtained by either method.

We then compared leukocyte infiltration into seven tumors and matched non-malignant tissue obtained from the periphery of the resected tissue. There was a significant increase in the infiltration of all leukocyte subsets into the tumors compared to minimal numbers in the non-malignant tissue.

**Conclusion:**

From these results we conclude that this method may be of considerable use for the enumeration of cells in tissues. Furthermore, since it can be performed by laboratory technical staff, less time input is required by the pathologist in assessing the degree of leukocyte infiltration into tumors.

## Background

A variety of clinical and pathologic evidence indicates that tumors can stimulate immune responses, such as the presence of mononuclear cell infiltrates, composed of T-cells[1], NK cells[2], and macrophages[3] in many different tumors.

In the past few decades, a number of studies have established correlations between prognosis and the degree of leukocyte (lymphocytes[4]; dendritic cells[5]; cytotoxic T-cells[6,7]; gamma/delta T-cells[8]; tumor infiltrating lymphocytes[9]; and monocytes[10]) infiltration in a variety of cancers. However, in all of these studies, specific staining was performed on relatively thin tissue sections. In addition, labeled leukocytes were manually counted in one or more random areas of each tumor section. Since the leukocytes are distributed in a three-dimensional volume of tissue, manual counting on thin tissue sections may not be truly representative of the actual numbers and spatial distribution of cells within the tissue.

Several microscopy and image analysis techniques have been developed for the *in vitro *and *in vivo *three-dimensional quantification of antigens in tissues, including deconvolution microscopy[11], stereomicroscopy[12,13] (often used with deconvolution or Confocal microscopy (CLSM) for thicker tissue sections[14]), or CLSM[15,16].

In deconvolution microscopy light from all planes of focus is collected, usually via a digital or video camera, and image slices are recorded onto a computer. Since light is collected from the whole depth of the specimen at each focal plane, each image appears blurred, or convolved. Before image analysis can be performed, the images need to be deblurred, or deconvolved to render a sharp image. Depending on the thickness of the specimen this process can be very time consuming.

Stereomicroscopy, a technique mainly used for dissection and tissue manipulation has experienced a resurgence in recent years for the 3-D analysis of many proteins and gene structures in tissues[17]. Stereomicroscopes have a long working distance and, using a dual light path, can generate 3D images over a large depth of field. However, for thicker specimens, stereomicroscopy is often coupled with deconvolution or CLSM to improve image clarity.

In terms of speed of data acquisition, CLSM is probably the most advantageous microscope technology for the production and analysis of 3D samples. CLSM refocuses fluorescent light onto a pinhole which excludes up to 95% of light from outside of the focal plane, producing clear images, even in tissue up to 100 μm thick. One apparent major disadvantage of CLSM is the initial equipment cost. However, in laboratories with a high sample throughput, this cost may be offset by its efficiency over time. This is especially important in pathology, where many specimens are analyzed and the data is required for diagnosis and prognostic determination as quickly as possible.

Therefore, this study sought to design an image based method for the calculation of leukocyte numbers in thick tissue sections (20 microns) using three-dimensional laser scanning confocal microscopy.

## Results

### Indirect immunofluorescence staining for leukocyte markers

**Figure 1:**
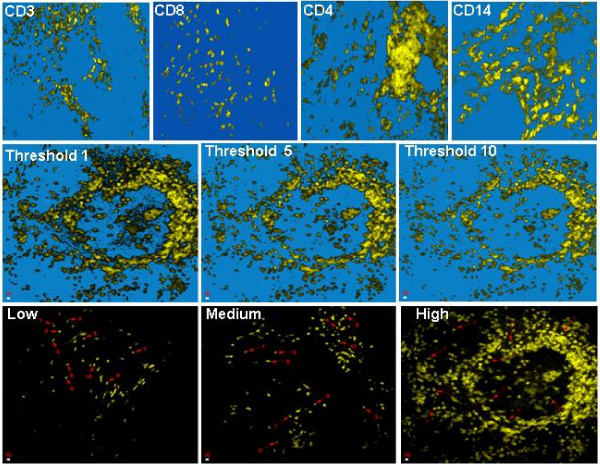
(top row) Indirect immunofluorescence detection of CD3 (T-cells), CD8 (cytotoxic/suppressor T-cells), CD4 (Helper/inducer T-cells) and CD14 (monocyte/macrophage) markers in 20 μm frozen tissue sections from an ovarian tumor. (middle row) Image thresholding on a high CD8 cell infiltration into an ovarian tumor. At a threshold of 1 noise can still be seen, which is removed at threshold 5. At a threshold of 10, significant data loss is observed. (bottom row) Ten cells from low, medium or high leukocyte infiltrate image stacks were chosen (arrows) which were entirely within the volume scanned, and their antigen volume calculated. The mean cell volume was divided into the total fluorescence volume at the selected threshold to enumerate leukocytes.

Indirect single immunofluorescence staining for four leukocyte markers: CD3 (T-cells); CD4 (helper T-cells); CD8 (suppressor/cytotoxic T-cells) and CD14 (monocyte/macrophage), was performed. Figure 1 (top row) shows representative look-through projections (from 20 optical slices) for each antigen. In some tissue sections cells could be easily distinguished because of their separation and even distribution in the tissue. However, in many tissue sections, clumps of cells were observed, which increased in size and distribution with increasing cell infiltration.

### Effect of threshold value on cell count

In order to calculate the volume of tumor occupied by a fluorescent antigen, a threshold was applied to the image stack to include only antigen-specific fluorescent signal. In addition to background (black or no fluorescence) removal, a threshold can be used to remove any residual noise resulting from setting the PMT to just include control fluorescence. The higher the threshold is set, the more fluorescence data are lost. To determine the effect of threshold setting on cell counts, three image stacks containing low, moderate or high numbers of cells were taken and the cell numbers calculated at ten threshold levels. Figure 1 (middle row) shows a representative high cell infiltration (high) image (derived from an image stack (CD8 antigen) at three of the ten thresholds examined. At a threshold of 1, background noise can still be clearly seen in the image, but is not present in subsequent images. At a threshold of 5, background noise was minimized, whereas at threshold 10 significant data loss was apparent.

At any particular threshold, the cell number was determined by division of the total antigen volume by mean cell volume. To determine the mean cell volume, ten cells from each image stack were seeded to calculate the volume of all contiguous fluorescence (cell volume) above the chosen threshold. Figure 1 (bottom row) shows representative projections of low, medium and high cell distributions indicating (arrows) the ten cells chosen. Each cell was checked to be separate from other cells, and to be entirely within the volume scanned.

**Figure 2:**
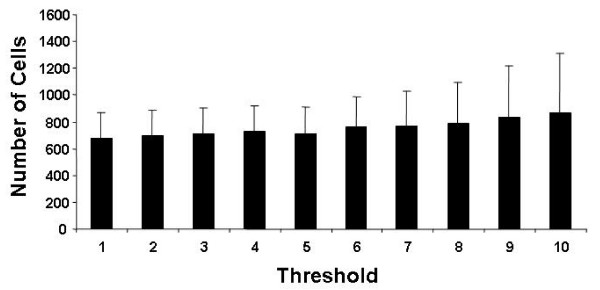
Effect of threshold value on cell count. Ten cells were selected from a CD3 image stack whose volumes were completely within the 20 optical slices scanned. The total tumor volume occupied by CD3 antigen was calculated at ten threshold values (1–10). The seeded volume of each of the ten selected cells was also calculated at the same threshold values. The total number of cells in the image stack was then determined by dividing the mean cell volume (of the ten cells) into the total CD3 volume at each threshold. Results are expressed as number of cells (+/- SD) at each threshold level. Cell counts remained relatively constant at lower thresholds, but gradually rose at higher thresholds.

Tables 1, 2 and 3 show the calculated volumes (in μm^3^) for image stacks containing low, medium, and high leukocyte infiltration respectively. The mean Cell Antigen Volume (CAV) for the 10 cells is shown at each threshold, as well as the standard deviation of the Cell Antigen Volume (SD CAV), the number of cells calculated for the image stack at each threshold, and the Total Antigen Volume (TAV) in the image stack at the corresponding threshold value are also shown. At all levels of cell infiltration the cell count remained relatively constant, because the same threshold was used to calculate the total antigen volume and the individual cell volume. At higher cell infiltration, a larger variation in calculated cell number was observed, but was still considerably lower than the variation from manual counting. The relatively constant relationship between threshold and calculated cell number is further illustrated in figure 2, which shows the effect of threshold (removal of grey scales between 1 and 10) on the resulting cell counts (cell number (bar) and SD (whisker) for the high cell infiltration image stack. Since total antigen volume and mean cell volume were both similarly affected by changes in threshold, their ratio (number of cells) also remained relatively constant at all thresholds tested. However, an increase in calculated cell number was observed in the high image stack at thresholds above seven, and was reflected in a larger standard deviation. It was not considered that the lower or higher threshold values would be selected by an operator for calculation, since obvious noise or data loss are apparent in the image stacks. To test this further, we asked 10 laboratory personnel (who had not previously performed thresholding) to threshold the three image stacks at a threshold level which they considered best maximized noise reduction, and minimized data loss. All personnel chose thresholds between 3 and 7 (mean 4 ± 1).

**Table 1:** Computer generated volumes for 10 individual cells (rows) after thresholding of grey scales 1–10 (columns) from an image stack with a low cell infiltrate. The mean Cell Antigen Volume (CAV) for the 10 cells is shown at each threshold, as well as the standard deviation of the Cell Antigen Volume (SD CAV), the number of cells calculated for the image stack at each threshold, and the Total Antigen Volume (TAV) in the image stack at the corresponding threshold value.

**Low Cell\Threshold**	**1**	**2**	**3**	**4**	**5**	**6**	**7**	**8**	**9**	**10**
**1**	790	676	581	482	336	283	252	206	174	138
**2**	424	363	308	255	178	148	120	104	85	65
**3**	677	575	520	445	332	287	247	190	161	140
**4**	741	663	596	533	425	376	329	286	244	218
**5**	504	451	411	382	323	298	274	257	232	210
**6**	836	759	711	650	561	524	483	455	424	401
**7**	688	657	624	589	520	495	463	431	401	371
**8**	854	810	747	680	599	558	530	502	476	461
**9**	434	396	358	330	268	249	230	214	196	176
**10**	875	795	730	665	571	525	480	441	397	365
										
**Mean CAV (μm3)**	**682**	**614**	**559**	**501**	**411**	**374**	**341**	**309**	**279**	**254**
**SD CAV (μm3)**	**172**	**163**	**157**	**148**	**145**	**142**	**138**	**137**	**134**	**134**
**Number of Cells**	**168**	**164**	**160**	**159**	**155**	**153**	**151**	**150**	**149**	**149**
										
TAV (μm3 * 10E6)	**0.11**	**0.10**	**0.09**	**0.08**	**0.06**	**0.06**	**0.05**	**0.05**	**0.04**	**0.04**

**Table 2:** Computer generated volumes for 10 individual cells (rows) after thresholding of grey scales 1–10 (columns) from an image stack with a medium cell infiltrate. The mean Cell Antigen Volume (CAV) for the 10 cells is shown at each threshold, as well as the standard deviation of the Cell Antigen Volume (SD CAV), the number of cells calculated for the image stack at each threshold, and the Total Antigen Volume (TAV) in the image stack at the corresponding threshold value.

**Medium Cell\Threshold**	**1**	**2**	**3**	**4**	**5**	**6**	**7**	**8**	**9**	**10**
**1**	641	557	481	418	359	295	228	182	147	118
**2**	787	710	641	583	545	502	458	424	399	354
**3**	568	517	456	419	397	361	330	308	286	263
**4**	433	370	326	281	242	211	175	138	117	98
**5**	469	428	376	336	316	286	272	251	236	214
**6**	649	510	466	433	388	369	338	313	291	273
**7**	509	489	402	359	325	298	265	237	214	195
**8**	459	409	374	339	304	285	263	243	227	212
**9**	658	583	536	489	453	416	384	356	326	302
**10**	642	582	527	473	431	395	364	324	298	272
										
**Mean CAV (μm3)**	**582**	**516**	**458**	**413**	**376**	**342**	**308**	**278**	**254**	**230**
**SD CAV (μm3)**	**113**	**100**	**94**	**88**	**87**	**83**	**83**	**84**	**84**	**80**
**Number of Cells**	**86**	**85**	**84**	**83**	**80**	**81**	**81**	**81**	**80**	**81**
										
TAV (μm3 * 10E6)	**0.05**	**0.04**	**0.04**	**0.03**	**0.03**	**0.03**	**0.03**	**0.02**	**0.02**	**0.02**

**Table 3:** Computer generated volumes for 10 individual cells (rows) after thresholding of grey scales 1–10 (columns) from an image stack with a high cell infiltrate. The mean Cell Antigen Volume (CAV) for the 10 cells is shown at each threshold, as well as the standard deviation of the Cell Antigen Volume (SD CAV), the number of cells calculated for the image stack at each threshold, and the Total Antigen Volume (TAV) in the image stack at the corresponding threshold value.

**High Cell\Threshold**	**1**	**2**	**3**	**4**	**5**	**6**	**7**	**8**	**9**	**10**
**1**	1741	1248	977	696	539	461	340	262	198	162
**2**	1541	1000	733	577	458	386	325	286	236	191
**3**	1618	1071	801	597	413	334	253	176	116	79
**4**	1951	1261	900	675	504	399	317	243	197	146
**5**	1484	1098	818	638	475	369	287	223	163	123
**6**	2402	1593	1169	893	695	559	458	382	288	243
**7**	2621	1853	1371	1070	856	689	586	473	386	319
**8**	1016	739	584	466	405	310	236	169	111	77
**9**	1951	1393	1046	830	705	561	479	401	329	279
**10**	2439	1578	1116	772	537	388	289	209	140	109
										
**Mean CAV (μm3)**	**1876**	**1283**	**951**	**721**	**559**	**446**	**357**	**282**	**217**	**173**
**SD CAV (μm3)**	**500**	**329**	**232**	**175**	**147**	**121**	**113**	**103**	**93**	**84**
**Number of Cells**	**685**	**700**	**714**	**735**	**716**	**762**	**772**	**796**	**847**	**874**
										
TAV (μm3 * 10E6)	**1.29**	**0.90**	**0.68**	**0.53**	**0.40**	**0.34**	**0.28**	**0.22**	**0.18**	**0.15**

There was considerable variability in the cell counts performed by the laboratory personnel for the low (59 cell difference), medium (98 cell difference) and high (300 cell difference) projections (Table 4).

**Table 4:** Manual leukocyte counts (Mean cell number, SD and range) for projections containing low, medium and high cell density.

Projection	Mean	SD	Range
Low	96	18	68–127
Medium	175	19	153–251
High	924	111	824–1124

Table 5 shows the mean, SD and range for the computer calculated cell numbers for the same low, medium and high cell density projections at ten threshold values. The ranges were smaller than the equivalent manual counts with cell differences remaining within 6 cells (low), 22 cells (medium) and 189 cells (high). If the manual threshold selection between 3 and 7 is also taken into account, these differences are further reduced to 3 cells (low; 82 ± 2), 9 cells (medium; 156 ± 4) and 79 cells (high; 793 ± 37).

**Table 5:** Computed leukocyte counts (Mean cell number, SD and range) for projections containing low, medium and high cell density.

Projection	Mean	SD	Range
Low	82	2	80–86
Medium	156	7	152–177
High	760	63	685–874

**Figure 3:**
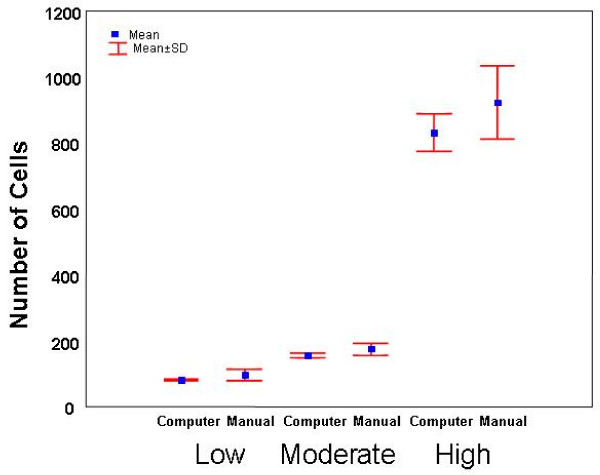
Comparison between manual counting and calculated leukocyte number in image stacks containing low, medium and high cell density. Manual counts were slightly higher than automated for the high projection. There was no significant difference (P > 0.05) between procedures for any of the three projections counted.

Figure 3 shows the mean and standard deviations for manual threshold selection compared to the computed method for the low, medium and high cell distributions shown in figure 1. No significant differences in cell count were observed between methods. However, the mean manual count for the high cell projection was slightly higher than for the calculated value (using the mean of ten thresholds), and significantly higher (P < 0.05) when the calculated results were adjusted between thresholds 3 and 7, reflecting a tendency for visual overestimation of cell number.

### Comparison between calculated and manual leukocyte counts in tumors

**Figure 4:**
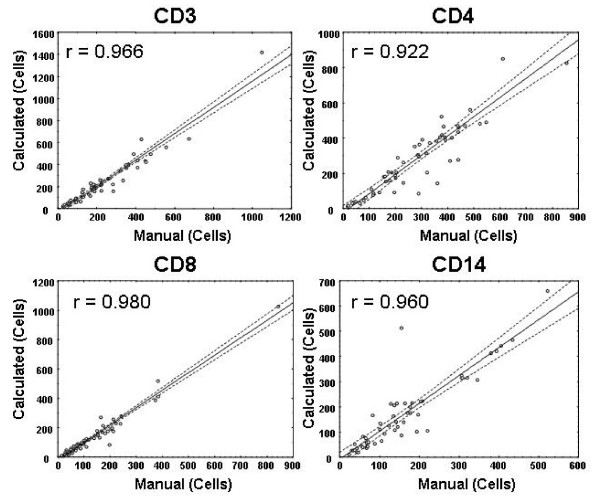
Correlation between manual and calculated cell enumeration in image stacks from twelve tumors for CD3 (n = 59), CD4 (n = 56), CD8 (n = 60) and CD14 (n = 56) in twelve tumors, where n is the number of image stacks processed. The two methods were highly correlated (r > 0.922) for all cell types tested.

The number of cells in each of the four leukocyte subsets in twelve tumor samples was analyzed. Approximately five image stacks per tumor were quantified for cell number: In some cases an image stack was rejected because it did not meet the criteria for inclusion (i.e. tissue was not present within the entire field of view by phase contrast observation). The number of image stacks (n) quantified for each antigen from the twelve tumors was, thus: CD3 (n = 59), CD4 (n = 56), CD8 (n = 60) and CD14 (n = 56). The number of cells in each image stack was determined directly by the computed method (Figure 4) and 3-D look-through projections were made for manual counts. Statistically significant (P < 0.01) correlations were observed in all cases.

### Comparison of leukocyte counts between tumor and non-malignant tissue

**Figure 5:**
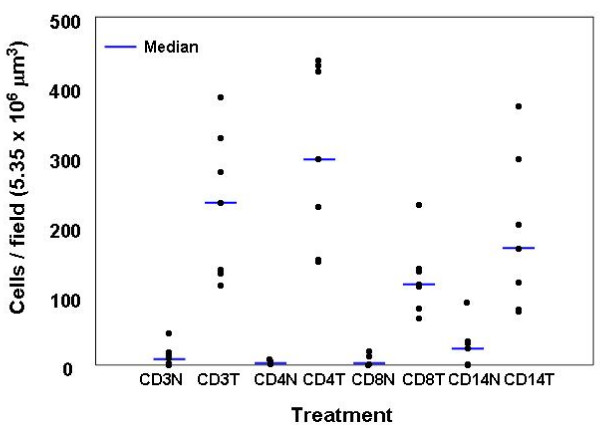
Comparison of leukocyte infiltration between seven tumors and donor matched non-malignant tissue. Results are expressed as individual values for each tumor and the median. Very few leukocytes were observed in non-malignant tissues. In contrast significantly higher leukocyte numbers (P < 0.05 in all cases) were observed in tumors for CD3, CD4, CD8 and CD14 cells. There was a large variation in tumor leukocyte numbers between tumors tested in this study.

Leukocytes expressing each of the four antigens were counted in seven tumors and donor matched non-malignant tissues, and their infiltration into these tissues compared (Figure 5). All of the non-malignant tissues showed low levels of infiltration of all leukocyte subtypes tested. In contrast, significantly increased (P < 0.05 compared to matched non-malignant tissue using the Wilcoxon signed rank test for paired non-parametric analysis) infiltration of all leukocyte subtypes was observed in corresponding tumor tissues.

## Discussion

We have designed a method for the enumeration of leukocytes in three dimensions using confocal laser scanning microscopy and image analysis software. The method has several advantages over visual assessment of leukocytes in hematoxylin and eosin stained tissue sections. Specific immunostaining for the leukocyte subtypes in thick tissue sections enables the assessment of the relative numbers of several leukocytes subtypes within a single specimen. Furthermore, refinement of the method by multiple immunostaining using different fluorophore tagged secondary antibodies would show the relative distribution of leukocytes within the same three dimensional volume of tissue. Thick (20 μm) cryosections are less difficult to cut than thin tissue sections (4–5 μm) typically used for histological analysis. Using thick tissue sections, CLSM can optically image thin slices at different focal planes within the tissue, which can be reconstructed to generate a three-dimensional stack. Enumeration of leukocytes in three-dimensional volumes is likely to be more representative of the true distribution of cells in the tissue, since their distribution may vary between image planes. A thin tissue section only represents one image plane for the pathologist.

Image thresholding removes voxels representing background (black) and low level non-specific staining. By choosing an appropriate threshold, the volume of tissue occupied by specific antigen can be calculated and expressed as a percentage of the total volume scanned[15]. Manual threshold selection is subjective and the results may vary if too much or too little threshold is applied. However, in the present application, the mean cell volume is also calculated at the same threshold as the total fluorescence volume. Thus, the calculated number of cells in each image stack remains relatively constant over a number of possible threshold choices (Figure 2). In fact, ten people who had not performed thresholding before chose thresholds between three and seven. When calculated between these thresholds, this represented a variation of only 79 cells (759 – 838) in the image stack with high CD8 cell infiltration. This variation was considerably lower than the 300 cell difference obtained when ten personnel manually counted the cells in a projection generated from the same image stack (716– 1124 cells; mean 924 ± 111). Although the two methods of counting were not significantly different (at 10 threshold levels), there appeared to be a tendency for manual overestimation of cell numbers, which became statistically significant when the calculated results were adjusted between three and seven (the range of thresholds chosen by ten personnel). These results demonstrated a tendency to manually overestimate the number of cells at higher densities in the tissues. At lower levels of cell infiltration the manual counts compared much better. For the twelve tumor samples tested, the two methods were highly correlated for all four antigens tested (Figure 4).

Using the calculated method, we then compared the leukocyte numbers in seven tumors with matched peripheral "non-malignant" tissue resected by the pathologist from the periphery of the tumor. It should be noted that although these tissues were assessed by the pathologist to be non-malignant, they may not necessarily be normal because of their proximity to the respective tumors. All non-malignant tissue sections showed minimal leukocyte numbers indicating a low level of infiltration (Figure 5). A larger variation in leukocyte numbers was observed for all cell types tested in the malignant tissues, but all tumors showed considerably increased leukocyte infiltration compared to their associated non-malignant tissue. The large variability in leukocyte infiltration into tumors may have been due to differences in the type and stage of disease in each case. Since the main purpose of this work was to compare methods of leukocyte enumeration, these factors were not taken into account.

This methodology is not restricted to the image analysis package we used to obtain the data reported in the present work. For example, we have recently adapted the method to ImagePro Plus (Media Cybernetics, Silver Spring, MD) with its plugin 3D Constructor. Many other commercially available and free packages such as NIH Image and Image J are available which will adequately perform thresholding, seeding and volume calculations without the need for special programming. In addition, three-dimensional image stacks can also be obtained from less expensive deconvolution or stereoscopic based microscopy systems which, although more time consuming, may be better suited to the budget of smaller pathology units.

## Conclusion

These data clearly demonstrate that enumeration of leukocytes using image analysis is a robust and rapid methodology which can be performed by relatively inexperienced laboratory staff. In addition, three dimensional image analysis overcomes the tendency to overestimate the number of cells in tumors with a high degree of leukocyte infiltration. Data obtained from clinical samples may help clinicians to establish the extent and type of immune response ongoing in the tumor, and thereby assist in both diagnosis and therapeutic strategy for individual patients.

## Methods

### Tumors and matched non-malignant tissues

For the development of thresholding and counting methodology, twelve freshly resected tumors comprising vulvar carcinoma (1); peritoneal carcinoma (2); endometrial carcinoma (2); neuroblastoma (1) and ovarian carcinoma (6) were obtained from the histopathology department at Florida Hospital in 30 ml universal containers (Barloworld Scientific, Staffordshire, UK) containing 10 ml RPMI 1640 tissue culture medium (Gibco, Grand Island, NY). The tissues were then transferred to 15 ml cryostorage vials (Nalge Company, Rochester, NY) and frozen in liquid nitrogen until required.

For experiments comparing malignant and non-malignant leukocyte infiltration, freshly resected tissue was obtained from seven patients. Representative (as assessed by a pathologist) pieces (1–2 cm) of endometrial carcinoma (1); ovarian adenocarcinoma (5) and neuroblastoma (adrenal; 1) and matched peripheral non-malignant tissue were obtained and treated as described above.

### Antibodies

Primary mouse anti-human monoclonal anti CD3 (IgG1), CD4 (IgG1), CD8 (IgG1), CD14 (IgG2a), and isotypic antibodies (murine IgG1 and IgG2a, were obtained from Beckman Coulter (Miami, FL). Oregon Green-labeled goat anti-mouse IgG (secondary antibody) was purchased from Invitrogen (Eugene, OR).

### Sectioning and immunofluorescence staining

Tumors were removed from liquid nitrogen, placed in the cryostat, left for 30 minutes to equilibrate at -20°C, placed onto cryostat tissue holders and embedded in mounting medium (CRYOform, International Equipment Company, Needham, MA). Serial 20 μm tissue sections were cut, placed on microscope slides, and allowed to air dry. Tissue sections were fixed in acetone/methanol (1:1) for 5 min, and washed three times using phosphate buffered saline (PBS; Gibco), with 5 minutes between washes. The slides were incubated with normal goat serum (10% in PBS; 100 μl vol) for 30 minutes, and washed in PBS as described above. One hundred microliters (containing 1 μg IgG) of primary antibody (CD3, CD4, CD8 or CD14) or isotypic IgG were applied to appropriate slides for one hour at room temperature in a humidified chamber. The slides were again washed three times with PBS and 100 μl of secondary antibody (containing 1 μg IgG) added to all slides except the blank (autofluorescence control), which was left in PBS. After a further three washes in PBS, coverslips were attached to the slides using Aqua-mount (Lerner Laboratories, PA).

### Confocal laser scanning microscopy

Slides were examined using a Multiprobe 2010 confocal laser scanning microscope (CLSM; Molecular Dynamics, Sunnyvale, CA), equipped with a Nikon Plan-Apo 20x (NA 0.75) air objective. Initially, slides were checked for specific antigen staining by comparing them to their isotype, second antibody, and autofluorescence controls. The photomultiplier tube (PMT) of the CLSM was set to just include light from the appropriate isotype control. Using the same PMT setting, five areas of tissue on each antibody labeled slide were serially scanned for Oregon Green fluorescence (areas were chosen using the halogen lamp alone to ensure that areas of high or low fluorescence were not inadvertently "selected"). For each field of view, a series of twenty serial optical slices, 0.6 μm apart, were scanned (*i.e*. 100 optical slices per slide). Data was processed on a Silicon Graphics workstation for image analysis. After image analysis, images and data were transferred to CD for storage.

### Image analysis techniques

#### Image thresholding and masking

**Figure 6:**
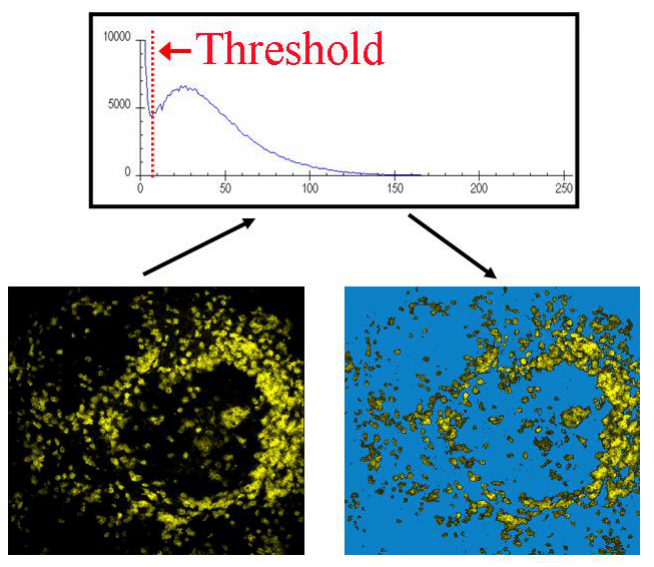
Illustration of image thresholding. To separate antigenic staining from background and noise, a threshold is applied to remove grey scales below a desired level (in this case 8). The graph shows the fluorescence intensity profile of the complete image slice (derived from an image stack). A threshold (dotted red line) is placed to separate data from non-fluorescent background and additional noise. All pixels (voxels in the complete stack) are then used for subsequent calculations.

In CLSM, fluorescent light is directed to a photomultiplier tube (PMT) which converts the light into electrical signals. As the laser scans across the optical field of view, fluorescent light of varying intensities is received by the PMT, and its emission voltage is digitally sampled (typically 512, 1024 or 2048 times per line of laser scan, depending on the resolution required). At each sampling point the intensity (voltage) is assigned to one of 256 grey scales (represented as "brightness" of the final image) ranging from 0 (black, no fluorescence) to 255 (white or maximum fluorescence recordable). Therefore, in CLSM, the PMT is set so that the range of fluorescent intensities within the sample fits within the grey scale range (which can be converted to any user defined color palette, using a look-up-table, if desired) so that varying intensities can be differentiated from each other. The final image is represented by a range of grey scales, some of which represent data of interest, and some represent no fluorescence or unwanted data or noise. In this study, a threshold was applied at grey scales ranging from 1–10, and all grey scales below the threshold were set to zero, a process known as masking[18], leaving only data of interest for quantification. Figure 6 shows an original image (left), and the corresponding intensity profile above it. A threshold grey scale was applied to delineate between black + noise, and data of interest. After application of the threshold, these lower grey scales were masked to zero (removed from the image), leaving the image on the right (the masked voxels are shown in blue for clarity). This procedure was repeated for each slice in the image stack to yield a total volume scanned (in the present study an image stack of 20 slices, 0.6 μm apart, giving 5.35 × 10^6 ^μm^3^).

#### Calculation of antigen volume

After thresholding, the number of voxels containing data of interest (i.e. specific antibody fluorescence) can be calculated as a volume in the same way that the total volume scanned was calculated, giving the relative proportion of the total volume occupied by antigen according to the following:

(Total antigen volume/total volume scanned) * 100%

The percentage of tumor volume occupied by antigen is a semi-automated method for providing the pathologist with data regarding the antigen content in tumors, as previously described [15].

#### Seeded region-growing segmentation analysis

**Figure 7:**
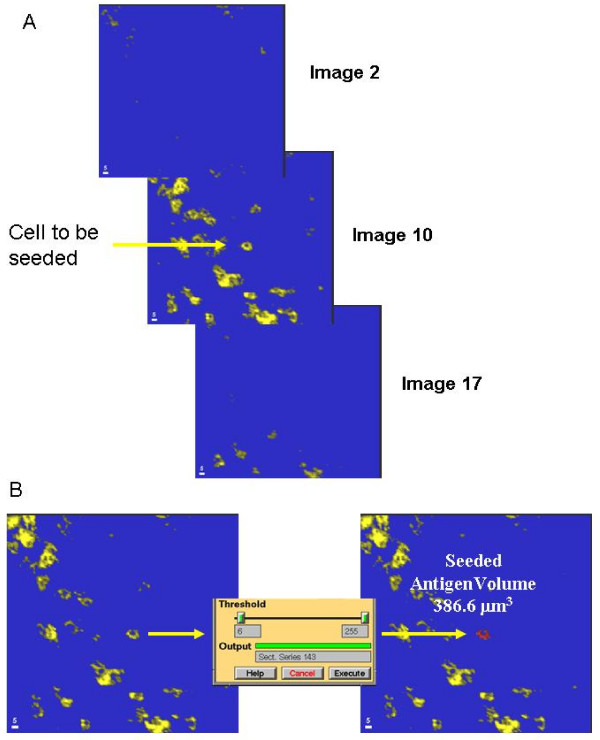
**A**. Three images from an image stack (nos. 2, 10 and 17 in a sequence of 20) showing a cell whose volume is entirely occupied within the volume scanned, since the cell body is not visible in ether images 2 or 17. **B**. seeding of the cell observed in A. using a mouse pointer a seed point is placed within the thresholded selected cell. A seeding region-growing segmentation algorithm is then applied. All contiguous fluorescence above the threshold is accumulated to derive the cell antigen volume.

Since this study investigated leukocyte infiltration into tumors, further image analysis can be performed to calculate the actual number of cells in each 3D volume. This is achieved by a process known as seeded region-growing segmentation analysis (seeding), which is a common function of many commercially available and free image analysis packages. After the application of a threshold to remove background and noise, many individual cells can be observed, as well as clumps of cells in heavier infiltrates. Prior to seeding, it is necessary to ascertain that the cell to be measured lies entirely within the volume scanned (if some part of a cell lies outside of the volume scanned, an erroneous cell antigen volume will be calculated) Figure 7A shows 3 slices from a 20 slice image stack. The cell to be seeded is observed in slice 10, but not in slice 2 or 17, confirming that it lies entirely within the volume scanned. This is a simple operation, since most image analysis packages allow for sequential views through the slices of an image stack.

Having ascertained that the cell is contained within the scanned volume, a mark or seed is placed on any part of the stained cell (using a mouse pointer) and the segmentation software calculated the volume of all contiguous voxels of fluorescence above the threshold. The region-growing stops when the threshold grey scale is reached. Figure 7B (left) shows the single cell chosen in Figure 7A. After seeding the cell (right) is falsely colored to show the extent of the region growth, and the cell antigen volume is automatically calculated.

This procedure was performed on every cell seeded in this study.

#### Enumeration of leukocyte infiltration

To enumerate leukocytes, ten isolated cells in each field of view were seeded and the mean of their volumes recorded. The total number of cells/field was determined by: mean cell antigen volume/total antigen volume

#### Composite projections used for manual counting

Using Imagespace™ software (Molecular Dynamics, Sunnyvale, CA), a look-through projection (i.e. the twenty optical slices were reconstructed to produce an image which appears to have been obtained from a transparent specimen by a lens with a large depth of focus) of each image stack was generated for each field of view. Look-through projections average the voxel intensities of exactly registered (directly in-line vertically through the stack) voxels from all slices in the image, and interpolate the data from the spaces within the slices. These processes generate an image which appears to be a composite of all the slices in the stack. The projections were printed and the cells manually counted.

### Comparison between calculated and manual cell counts in tumors

To compare calculated and manual cell counts in tumor tissue sections, ten cells were chosen from each slide which were entirely within the volume scanned, and the mean cell volume determined by seeding, as described above. The number of cells in each image stack was then calculated by dividing the total antigen volume by the mean cell volume. Using Imagespace™ software, the volume of tissue in each image stack was calculated. Since all scanning conditions were kept constant, a constant volume of tumor was scanned in each image stack (5.35 × 10^6 ^μm^3^). A threshold was set to exclude zero fluorescence (black) and any residual background staining, and the total volume of scanned tumor occupied by stained cells was determined.

A look-through projection of each image stack was also printed and the cells manually counted. The manual counts were compared to the computer generated cell numbers using Student's t-test for dependent variables.

### Effect of threshold value on cell count

To determine the effect of threshold level on cell count, three image stacks were selected (two CD3 and one CD8 series) which contained low (relatively few, well separated cells), moderate (well separated cells and some cell clumps) and high (few clearly separated cells and many clumps) cellular density. Ten cells were chosen from each image stack which were entirely within the volume of tissue scanned, and their volumes determined at 10 threshold levels from grey scale 1 to grey scale 10, using ImageSpace™ software. The total tumor volume occupied by fluorescent antibody was also calculated at each threshold. The total number of cells in the image stack was then calculated by dividing the mean cell volume (of the ten cells) into the total antigen volume at each threshold.

### Manual cell counts

Look-through projections were printed, and the number of cells in each projection manually counted. To assess operator variability for manual counting the printed projections were counted independently by 10 laboratory personnel. The two counting methods were then compared using a Student's t-test for dependent variables.

## Authors' contributions

**JB **designed the overall project, performed sample acquisition, imaging, analysis, and wrote the majority of the text.

**BW **performed data analysis and statistics, and assisted in manuscript preparation.

**AA **performed tumor sectioning and manual cell counting and statistics.

**JF **liased with clinicians and pathologists to obtain samples, directed the study, and assisted manuscript preparation.
